# Stability Enhancement of Anthocyanins from Blackcurrant (*Ribes Nigrum* L.) Pomace through Intermolecular Copigmentation

**DOI:** 10.3390/molecules27175489

**Published:** 2022-08-26

**Authors:** Ezzat Mohamad Azman, Nurhayati Yusof, Afroditi Chatzifragkou, Dimitris Charalampopoulos

**Affiliations:** 1 Department of Food and Nutritional Sciences, University of Reading, Whiteknights RG6 6AH, UK; 2 Faculty Food Science and Technology, Universiti Putra Malaysia, Serdang 43400, Selangor, Malaysia; 3 Faculty of Bioresources and Food Industry, Universiti Sultan Zainal Abidin, Besut Campus, Besut 22200, Terengganu, Malaysia

**Keywords:** intermolecular copigmentation, anthocyanin stability, pH, color retention, storage, antioxidant activity

## Abstract

Intermolecular copigmentation denotes the interaction between colored anthocyanins and the colorless copigment, which is not bound covalently to the anthocyanin molecule. This is the first study to investigate the effect of intermolecular copigmentation on the stability of individual anthocyanins from dried blackcurrant pomace (DBP) using four pure phenolic acids as copigments (ferulic, caffeic, chlorogenic, and rosmarinic acid). Studies were performed at pH 3.0 and pH 6.0, with a copigment/anthocyanin extract molar ratio of 5:1, during storage at 20 °C. At both pH 3.0 and 6.0, rosmarinic acid showed the strongest hyperchromic and bathochromic effects (*p* < 0.05) on day 0. However, rosmarinic acid showed low stability during storage. At pH 3.0, chlorogenic acid and control samples were capable of maintaining very high levels of total anthocyanin stability during storage (*p* < 0.05). On the other hand, ferulic acid and control samples had the longest estimated half-life during storage at pH 6.0. Intermolecular copigmentation successfully increased the half-life, color retention, and antioxidant activity of the anthocyanin solution, with cyanidin-3-*O*-glucoside (C3G) exhibiting the highest stability at both pH values. Overall, anthocyanins from DBP, in combination with chlorogenic or ferulic acid, showed potential for use in commercial food applications.

## 1. Introduction

The phrase “no artificial colors/flavors” has been shown to influence consumers when buying foods and beverages [1]. This so-called “clean label” trend is due to the growing awareness of natural ingredients and as a way to avoid the potential side effects of the artificial substances used in synthetic food colorants [2,3]. Annually, rapid and continuous growth of 10% to 15% is expected in the production of natural food pigments worldwide [3]. Commonly used plant-derived food pigments include anthocyanins, carotenoids, betalains and chlorophylls. Both chlorophylls and carotenoids demonstrate moderate heat stability, while betalains and anthocyanins show moderate to low and high to moderate heat stability, respectively [4]. Anthocyanins, carotenoids, and betalains are mainly used in the production of beverages, confectioneries, and jelly-like products, while chlorophylls are added to citrus-based dry beverage mixtures [5].

Anthocyanins are natural products that could be considered candidates for the replacement of synthetic dyes, due to their bright, attractive colors (orange, red, and purple), high water solubility, and possible health benefits, including their antioxidative effects, the prevention of cardiovascular disease, and improved visual health [3,6,7,8]. Anthocyanins have been described as antioxidants and free-radical scavengers that can prevent or inhibit oxidation by donating electrons to those free radicals with unpaired electrons [9]. Sytar et al. [10] proved that the presence of anthocyanins in the plant parts of buckwheat is correlated with the presence of rutin content. However, the main challenges that need to be addressed before anthocyanins can be incorporated into food systems rely on the fact that as natural colorings, they have poor stability, present dull shades, and exhibit rapid color-fading when exposed to light, oxygen, high temperatures, pH, salt stress and enzymes such as polyphenol oxidases (PPO) and peroxidases (POD) [11,12,13].

**Figure 1:**
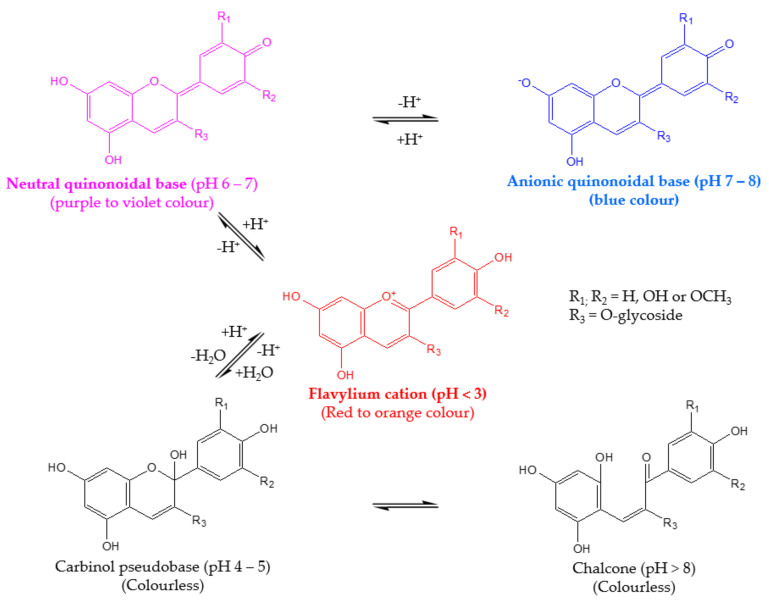
Effect of pH on the structural transformation of anthocyanins and the resulting color. Adapted from Houghton et al. [19].

A particular issue with anthocyanins is their sensitivity to changes in pH since anthocyanins lose their color at pH values higher than 3.0; therefore, this limits their use as natural colorants to appear only in acidic food products [14]. As shown in Figure 1, at a pH below 3, anthocyanins exist mainly as the very stable red flavylium cation form. As the pH increases from 4 to 5, the colorless carbinol pseudobase is generated due to the rapid hydration of the flavylium cation. Deprotonation of the flavylium cation at pH 6–7 exhibits the formation of a neutral quinonoidal base (with a purple to violet color), which leads to the formation of an anionic quinonoidal base at pH 7–8 (blue color) [15,16,17,18,19]. 

**Figure 2:**
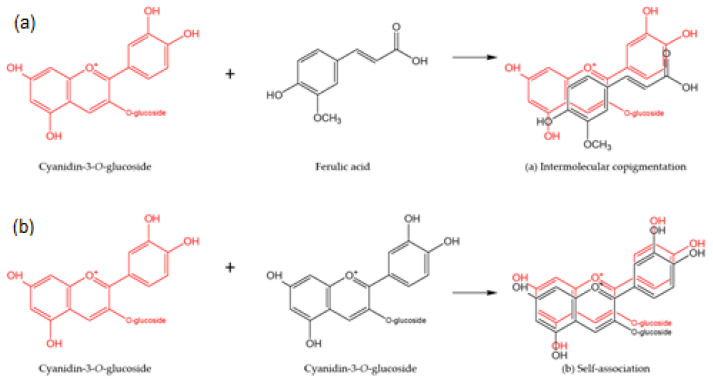
Examples of anthocyanin interaction. (**a**) Intermolecular copigmentation and (**b**) self-association. Adapted from Castañeda-Ovando et al. [24].

The stability of anthocyanins can be improved by copigmentation. The types of copigmentation reactions include intermolecular and intramolecular copigmentation, self-association, and metal complex formation [20]. The term “intermolecular copigmentation” is reserved for anthocyanins, where a pigment (anthocyanins) and a copigment (mainly phenolic acids and flavonoids) are mixed directly by non-covalent bonds in a solution [18] (Figure 2a). Another form of copigmentation is “self-association”, where two or more anthocyanin molecules are associated via stacking-like interactions; the mechanism was first suggested by Asen et al. [21] (Figure 2b). Hydrophobic and “π–π”-interactions between the electron-rich copigments and electron-poor flavylium ions that are present in the anthocyanin molecules protect the anthocyanin chromophore (in the flavylium cation form) against the nucleophilic attack of the water molecules, which may result in the formation of its colorless hydrated forms (chalcone or carbinol pseudobase). Non-covalent complexes between the copigments and anthocyanins form a parallel stacking phenomenon and not only exert a protective effect on anthocyanins but also contribute to the color stabilization of the system [22,23]. This results in the increased absorbance of the copigmentation complex (hypherchromic effect ΔA), whereas the wavelength of the maximum absorption shifts toward higher wavelengths (bathochromic shift, Δλ), which, in turn, increases the color intensity. 

The copigmentation reactions can improve the color stability of anthocyanins and increase their half-life, thereby making it possible for them to be commercially applied as natural colorants in food matrices. A considerable number of studies in this area have shown that intermolecular copigmentation is influenced by the types and origins of anthocyanins (e.g., strawberry, raspberry, lingonberry, and cranberry), the copigment structure and its concentration, the pH of the medium, temperature, storage conditions and the presence of organic co-solvents in the medium [25,26,27]. These studies have demonstrated that phenolic acids, flavanols, alkaloids, metals, and anthocyanins themselves (the latter through self-association) are potent copigments for copigmentation reactions. However, most of the previous works have been conducted with fruits that contain acylated anthocyanins, such as grapes and purple sweet potatoes, which are well known for their high stability compared to non-acylated anthocyanins [27,28]. 

**Figure 3:**
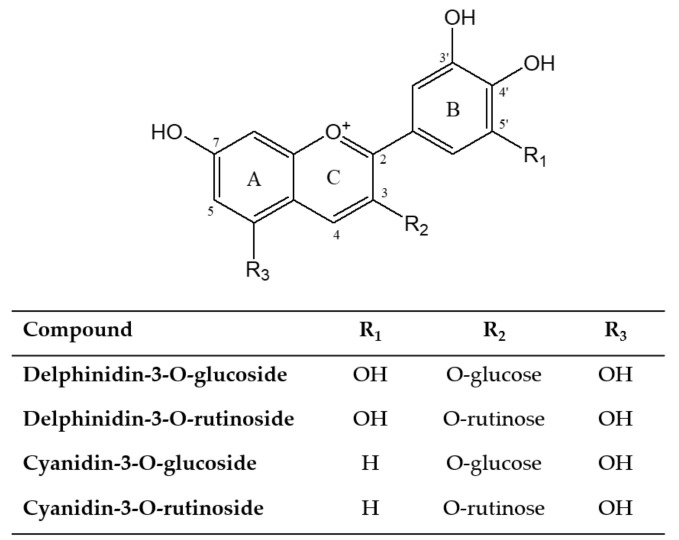
Chemical structures of anthocyanins sourced from DBP.

Annually, more than 300 tonnes of blackcurrant by-products are produced in the UK, containing high amounts of anthocyanins (14–18 mg/g) [29]. These by-products can be adopted relatively easily by the food industry and have been used as a natural food colorant for many years [7,30]. DBP constitutes four types of non-acylated anthocyanins, namely, delphinidin-3-*O*-rutinoside (D3R), cyanidin-3-*O*-rutinoside (C3R), delphinidin-3-*O*-glucoside (D3G) and cyanidin-3-*O*-glucoside (C3G) [29] (Figure 3). However, they are less stable than the acylated forms; thus, a reaction such as copigmentation must be carried out in order to increase their stability. 

Therefore, the overall aim of this work was to enhance our fundamental understanding of the intermolecular copigmentation reactions that take place between the anthocyanins present in a semi-purified extract and phenolic acids. Trouillas et al. [18] revealed that the hydroxycinnamates (caffeic, ferulic, chlorogenic, and rosmaric acids, etc.) were better copigments than hydroxybenzoic acid (gallic, benzoic, vanillic, and syringic acids, etc.). Until recently, no study has previously been conducted on the intermolecular copigmentation of each individual anthocyanin from DBP in strongly and weakly acidic buffer solutions. To achieve these aims, the study centered on two key objectives: (i) to investigate the effect of pH 3.0 and 6.0 on the intermolecular copigmentation of anthocyanins from DBP, in terms of the different structures of molecular anthocyanins with a number of phenolic acids (ferulic, caffeic, chlorogenic, and rosmarinic acids); (ii) to assess the stability of the copigmentation complexes during storage at 20 °C. 

## 2. Materials and Methods

### 2.1. Chemicals

All solvents and chemicals used for extraction, including methanol (99.9%), ethyl acetate (99%), and 1,1-diphenyl-2-picryl-hydrazyl (DPPH) were of analytical grade and were purchased from Sigma-Aldrich (Dorset, UK). Potassium sorbate, citric acid monohydrate, and anhydrous sodium phosphate were also purchased from Sigma-Aldrich (Dorset, UK). Hydrochloric acid (37%) was purchased from Fisher Scientific (Loughborough, Leicestershire, UK). 

Purified water, purified through a Purite reverse osmosis system (SUEZ Water Purification Systems Ltd., Thame, Oxon, UK), was used for buffer preparations, as well as for analyses. The anthocyanin standards of C3G (96%), C3R (96%), D3G (95%), and D3R (95%) were obtained from ExtraSynthese Ltd. (Genay, France). The copigments and ferulic, caffeic, chlorogenic, and rosmarinic acids were purchased from Sigma-Aldrich (Dorset, UK).

### 2.2. Preparation of Dried Blackcurrant Pomace (DBP) 

Dried blackcurrant pressed residues from a juice-manufacturing process were kindly supplied by A & R House (BCL) Ltd., (Bleadon, Weston-super-Mare, UK). The seeds, which were present in the residue samples, were separated by initially grinding the samples in a coffee blender and subsequently passing them through a 0.841 mm (20 mesh) sieve; the obtained fraction constituted the DBP fraction. The DBP samples were segregated in polyethylene bags and stored at −20 °C until further analysis.

### 2.3. Extraction of Anthocyanins from Dried Blackcurrant Pomace (DBP)

The anthocyanins were extracted from DBP (50.0 g) according to the protocol by Stevenson et al. [31], which involved steeping the DBP sample in 250 mL of 0.1% (*v*/*v*) HCl in methanol for 1 h. The obtained extracts were vacuum-filtered to separate the supernatants and solid residues, while the latter underwent a second extraction with 250 mL of fresh solvent for another 1 h. The extraction process was repeated twice more, then the supernatants were pooled together and evaporated under vacuum by a rotary evaporator to remove the methanol. Then, 100 mL of distilled water was added to the residue; this constituted the crude anthocyanin extract sample.

### 2.4. Purification of Anthocyanin Extract

The anthocyanin-containing extract was introduced into a Strata^®^ C18-U (55 µm, 70 Å, 500 mg/6 mL) cartridge (Phenomenex Ltd., Macclesfield, UK) after 5-fold dilution with distilled water. The extract was then washed with 12.0 mL of 0.01% (*v*/*v*) HCl in water and subsequently with 12.0 mL of ethyl acetate, in order to remove the non-phenolic compounds, such as sugars, as well as those phenolic compounds other than anthocyanins, such as flavonoids, respectively [32]. Finally, the anthocyanins were eluted with 0.01% (*v*/*v*) HCl in methanol, followed by evaporation to remove the solvents; then, 100 mL of distilled water was used to solubilize the residue. The purified anthocyanin-containing extract was lyophilized and stored at −20 °C until further analysis.

### 2.5. Preparation of Buffer Solutions

Phosphate-citrate buffer solutions of pH 3.0 and 6.0 were prepared according to the procedures of Sigma [33]. The pH of each buffer solution was adjusted with 5 M HCl or 5 M NaOH and measured with a pH meter (Mettler Toledo Seven Easy, UK). The pH meter was calibrated with the standard solutions of pH 7.0 and 4.0 (Mettler Toledo, Leicester, UK).

### 2.6. Pigmentation Reactions and Stability Studies 

#### 2.6.1. Copigmentation Reactions

The molar ratio of copigments to anthocyanins (expressed as C3G) that were present in the extract was set to 5:1, based on a previous study by Shikov et al. [34]. Four phenolic acids were used for the copigmentation studies: namely, ferulic, caffeic, chlorogenic, and rosmarinic acids. First, 22.5 mg and 75 mg of freeze-dried purified anthocyanins were added to 100 mL of pH 3.0 and pH 6.0 buffer solutions, respectively. The number of purified anthocyanins to be added was decided by preparing the corresponding anthocyanin buffer solution with a concentration of around 75 mg/g, as measured by the pH differential method [35]. Then, the weight of the copigments (phenolic acids) added to the buffer solutions was calculated to achieve a 5:1 molar ratio of copigment to the total anthocyanins in the extract. 

The reactions were conducted in 100 mL amber bottles with lids, containing 0.05% potassium sorbate (*w*/*v*), to inhibit potential microbial growth. The pH of the reaction mixture was adjusted to pH 3.0 or 6.0 using 5 M HCl or 5 M NaOH. The solutions were shaken at 150 rpm on an orbital shaker (VWR, Model 3500, Houston, Texas) for 30 min and left to rest for 2 h in the dark at room temperature to reach equilibrium [36]. Purified anthocyanin extract with no phenolic acids added was included and served as the control.

#### 2.6.2. Stability Studies

Following the reactions, the samples were kept in the dark at 20 °C. Samples were taken at regular intervals and analyzed until the time point when at least 50% of the anthocyanins were degraded. The stability studies were carried out in duplicate.

### 2.7. UV-Vis Spectrophotometry and Color Measurements

Spectrophotometric measurements of the control and intermolecularly copigmented samples were carried out using a UV-Vis spectrophotometer (Perkin Elmer Lambda 20, Waltham, MA, USA). The spectra were recorded in 1-cm path length disposable cuvettes at wavelengths from 400 to 700 nm. The copigmentation phenomena were related to a bathochromic shift, i.e., a shift of the maximum absorption wavelength (λ_max_), and a hyperchromic effect, i.e., an increase in the absorbance value (∆*A*) at λ_max_. Each spectroscopic measurement was performed in duplicate. The percentage of color retention at λ_max_ was used to evaluate the color degradation at the two different pHs over time and was calculated according to the following formula (Equation (1)):

(1) $$Color\;Retention\;\left(\%\right)=\frac{A_{t}}{A_{o}}\times 100$$

where *A*_0_ is the initial absorbance at time zero and *A*_t_ is the absorbance at time *t* [37].

The sample color was measured using a Hunter-Lab colorimeter (Hunter Lab, ColorQuest, Hunter Associates Laboratory, VA, USA) based on the three-color coordinates, L*, a*, and b* at D65 standard illuminant and 10° standard observer. The instrument was calibrated using a black card. Color was expressed in Hunter Lab units L* (lightness/ darkness; 0–100), a* (positive = redness/negative = greenness) and b* (positive = yellowness/negative = blueness). The total color difference (TCD) between any two samples was calculated according to the following formula (Equation (2)):

(2) $$Total\;color\;difference\;\left(TCD\right)=\left[{\left(L^{*}-L_{o}\right)}^{2}+{\left(a^{*}-a_{o}\right)}^{2}+{\left(b^{*}-b_{o}\right)}^{2}\right]1/2$$

where, *L*_o_, *a*_o_, *b*_o_ = blank values of the control sample (purified anthocyanin extract) at day 0.

Chroma (c) is the quantitative attribute of color intensity, while hue (ℎ) is the qualitative attribute of colors that are defined as reddish, greenish, yellowish, and bluish. The chroma and hue angle were calculated using the a* and b* values, according to Equations (3) and (4) below [38]:

(3) $$Chroma\;\left(c\right)=\sqrt[{}]{{\left(a^{*}\right)}^{2}+{\left(b^{*}\right)}^{2}}$$

(4) $$Hue\;angle\;\left(h\right)=ArcTan\left(\frac{b^{*}}{a^{*}}\right).$$

### 2.8. HPLC Analysis of Anthocyanins

HPLC analysis of the anthocyanin compounds was based on a method developed by Azman et al. [39] using a Waters 2695 Alliance HPLC system (Waters Corp., Milford, MA, USA) equipped with a Waters 2478 two-channel UV detector, two Waters 515 HPLC pumps, an auto-sampler, a column oven, and an online degasser. Analyses were carried out with a Purospher STAR RP18 end-capped column (250 mm × 4.6 mm i.d., particle size of 5 μm, Merck, Darmstadt, Germany) at 30 °C. The mobile phase consisted of 2% (*v*/*v*) of formic acid in water solution (solvent A) and 100% (*v*/*v*) methanol (solvent B). The gradient elution system was 15% (B) at 0 min, increasing to 35% (B) at 15 min and to 60% (B) at 30 min, reaching 80% (B) at 40 min. The flow rate was 1.0 mL/min and the injection volume was 20 µL. The duration of the analysis was 50 min.

The detection and quantification of the main anthocyanins present in the purified extract (D3R, C3R, D3G, and C3G) and the reaction samples were carried out at a wavelength of 520 nm, based on external standard solutions and their respective calibration curves.

### 2.9. Kinetic Study

The zero-order reaction rate constants (*k*) and half-lives (*t*_1/2_) describing anthocyanin degradation during storage were calculated using Equations (5) and (6):

(5) $$C_{t}=C_{o}-k^{*}t$$

(6) $$t_{1/2}=\frac{C_{o}}{2k}$$

where *C*_0_ is the initial anthocyanin concentration and *C_t_* is the anthocyanin concentration at time *t* [40].

### 2.10. DPPH Radical Scavenging Activity

Determination of the antioxidant activity using the DPPH radical scavenging activity was carried out according to the method of Azman et al. [29] with some modifications. Briefly, 100 μL of the sample was mixed with 2 mL of 0.15 mM DPPH reagent, which was prepared in methanol. The antioxidant activity was expressed as the percentage of DPPH scavenging. Duplicate measurements were taken and the mean values were calculated.

### 2.11. Statistical Analysis

All statistical analyses were conducted by a one-way analysis of variance (ANOVA). Tukey’s multiple range tests were employed, with a probability of *p* < 0.05. A linear Pearson correlation was also used to evaluate correlations between anthocyanin half-life and the percentage of color retention. The software used for the statistical analysis was Minitab V.19 (Minitab Inc., State College, PA, USA).

## 3. Results and Discussion

**Figure 4:**
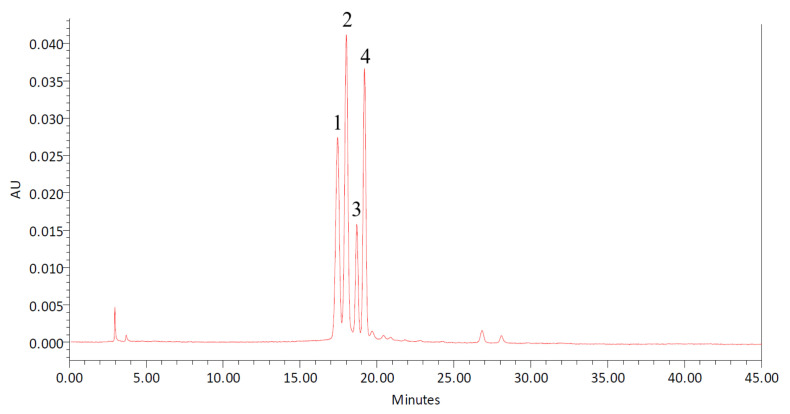
Typical HPLC chromatogram of DBP, showing the detected free anthocyanins at 520 nm. (1) Delphinidin-3-*O*-glucoside, (2) delphinidin-3-*O*-rutinoside, (3) cyanidin-3-*O*-glucoside, and (4) cyanidin-3-*O*-rutinoside.

The DBP extracts contained four main types of anthocyanins, namely, D3G, C3G, D3R, and C3R. According to the HPLC results (Figure 4), the anthocyanin content in the purified extracts was calculated as 9.0 ± 0.1 mg/g, corresponding to 88.1 ± 0.6% of purified anthocyanin recovery from the original DBP samples. Notably, due to their similar chemical characteristics such as polarity, phenolic compounds other than anthocyanins are difficult to separate in the HPLC column as they have identical retention times, which is in agreement with the findings by Mizzi et al. [41]. In this study, the crude extract was purified using solid phase extraction (SPE) on a C18 cartridge to eliminate impurities such as sugars and other flavonoid materials [42], to allow more thorough testing of the effect of copigmentation on the individual DBP anthocyanins.

### 3.1. Effect of Copigmentation on UV-Vis Absorption Spectra

#### Copigmentation Effect in Buffer Solutions at pH 3.0 and pH 6.0

The copigmentation of anthocyanins with phenolic acids in buffer solutions increased the maximum absorption wavelength (bathochromic effect, ∆λ_max_) and absorbance (hyperchromic effect, ∆*A*_max_), which, in turn, increased the color intensity of the anthocyanin solutions on the day of preparation (day 0), as shown in Table 1. At pH 3.0, rosmarinic acid had the highest percentage of hyperchromic effect (∆*A* = 20.2%) and bathochromic shift (∆λ = 6 nm), followed by ferulic acid (17.4%; ∆λ = 5 nm), chlorogenic acid (7.2%; ∆λ = 4 nm), and caffeic acid (7.7%; ∆λ = 3 nm), compared to the control (with no copigment) (Table 1a). When the copigment was another phenolic compound, the opposite charge of the compounds interacted to produce a charge-transfer complex formation or π–π interactions [43]. As the flavylium ion in the anthocyanin was positively charged, it became a suitable candidate for the formation of complexes via charge transfer with rich electron substrates [24], thus increasing the hyperchromic effect to the maximum absorbance [44]. 

Rosmarinic acid exhibits two aromatic ring systems, each substituted with two hydrogen bond donor/acceptor OH groups. Thus, rosmarinic acid may be able to form stronger π-π stacking interactions with anthocyanin molecules, thereby resulting in color enhancement, as observed in the current study. A similar pattern was demonstrated by Eiro and Heinonen [45] at pH 3.37, where rosmarinic and ferulic acids enhanced the color of the complex by 80% and 70%, respectively, on the day of preparation. Moreover, rosmarinic acid proved a better copigment than chlorogenic acid in diluted sweet potato concentrate at an acidic pH (0.9, 2.6, 3.6, and 4.6) [27]. 

According to Asen et al. [21] and Brouillard et al. [46], other than flavylium cation at pH 3.0, copigmentation can also occur at the neutral quinoidal base of the anthocyanins at pH 6.0. As shown in Table 1b, there was a significant (*p* < 0.05) increase in the percentage of hyperchromic effect (∆*A* = 73.8%) and bathochromic shift (∆λ = 11 nm) when using rosmarinic acid as a copigment, followed by chlorogenic acid (∆*A* = 49.8%). On the other hand, ferulic and caffeic acid had the lowest increase but were still higher compared to pH 3.0 (∆*A* = 31.9%; ∆λ = 1 nm and ∆*A* = 17.3%; ∆λ = 1 nm, respectively). The increase in the λ_max_ and *A*_max_ values suggested that copigmentation also occurred at pH 6.0 and the values were statistically more significant (*p* < 0.05) than in acidic media at day 0, which was in agreement with Boulton [47], who confirmed that copigmentation showed greater intensity in a higher pH with neutral quinonoidal bases. This is due to the fact that the color increase is maximal in a mildly acidic, almost colorless solution. On the contrary, the hydration equilibrium is no longer established at a low pH; no colorless forms are available for conversion into colored forms upon copigment addition [18]. Hence, in this study, higher hyperchromic and bathochromic effects were seen at pH 6.0 than at pH 3.0.

### 3.2. Copigmentation Stability during Storage

In the subsequent stability tests, the DBP anthocyanins-copigment complexes were kept at 20 ± 1 °C where the half-life and color stability of the anthocyanin solutions in buffer solution at pH 3.0 and 6.0 were evaluated until the anthocyanins reached 50% of the estimated half-life. Table 2a,b show the degradation kinetics of individual anthocyanins, as measured by HPLC compared to day 0, following zero-order reaction kinetics, with a correlation coefficient (R^2^) greater than 0.900. Studies have shown that the degradation of monomeric anthocyanins usually resulted in the formation of polymeric pigments (e.g., phenolic acid and aldehyde compounds), which is related to the degradation of the color [24,48].

#### 3.2.1. Effect on Anthocyanin Stability

As shown in Table 2a, chlorogenic acid and the control samples exhibited the highest half-life of anthocyanins at pH 3.0 (71.0 and 71.8 days, respectively), followed by rosmarinic acid, ferulic acid, and caffeic acid. On the other hand, at pH 6.0, ferulic acid and the control samples showed a significantly high (*p* < 0.05) estimated half-life (5.5 and 5.3 days, respectively), followed by chlorogenic, caffeic, and rosmarinic acid samples (Table 2b). The stacking of the phenolic acid occurred on the planar polarizable nuclei of the colored anthocyanin forms; in this study, chlorogenic acid on a flavylium cation at pH 3.0 or ferulic acid on the quinonoid forms at pH 6.0 prevents the nucleophilic attack of water or hydration at position 2 of the pyrylium nucleus [49,50]. These phenolic acids prevented the hydration of the flavylium cation or the neutral quinonoidal base on the anthocyanin structure from the colorless carbinol pseudobase formation, as shown in Figure 1, and consequently, stabilized the anthocyanin better than the other copigments. 

There are only a few studies that focused on the copigmentation effect in a neutral pH environment but without investigating the effect of copigmentation during storage. In this study, the estimated half-life at pH 6.0 was significantly lower than pH 3.0, which was in agreement with Mazza and Brouillard [50] and Brouillard et al. [51], where the flavylium cation bound phenolic copigments, such as flavonoids and hydroxycinnamic acid, slightly more strongly than the corresponding quinonoid bases. 

The observation that the control samples showed a longer half-life compared to some of the copigmentation samples can possibly be attributed to the fact that molecular complexes with other anthocyanins (self-association) are more stable compared to other colorless compounds (intermolecular copigmentation), as suggested by Trouillas et al. [18] (Figure 2). Self-association between anthocyanins was found to be stronger with an increasing number of hydroxyl or methoxy substituents on the B-ring [52]. In this study, cyanidin- and delphinidin-derived anthocyanins have two and three hydroxyl groups attached to the B ring, respectively. The addition of certain phenolic acids during the copigmentation process interrupts the association between self-stacking anthocyanins and accelerates anthocyanin degradation [53]. 

In addition, similar structural features make the intermolecular interactions between the flavylium cation and quinonoidal base forms of the anthocyanin or with copigments much easier [21]. Clearly, interactions between phenolic acids and anthocyanins are less efficient compared to those between the anthocyanins, based on the similarity of structural features. Therefore, the molecular stacking of the aromatic units of phenolic acids with the chromophore of anthocyanins demonstrated a lower ability to prevent hydration of the anthocyanidin nucleus, compared to anthocyanin self-stacking [53,54]. This mechanism might be why, except in the case of chlorogenic acid and ferulic acid, anthocyanin degradation proceeded faster with phenolic acids in this study. The interaction between anthocyanins and copigments also caused an increase in temperature (exothermic reaction) and led to the formation of colorless compounds that can cause the dissociation of copigmentation complexes [50]. 

According to the HPLC results at day 0, D3R appeared to be the most dominant anthocyanin in all samples at pH 3.0 (D3R (~34.5%) > C3G (~31.1%) > D3G (~21.2%) > C3R (~15.9%)). However, during storage, C3G exhibited the highest stability (*p* < 0.05) in all samples, with an estimated half-life of between ~90 and ~222 days. Moreover, there were no significant differences in the half-life between D3G, D3R, and C3R. The results obtained are in agreement with previous reports by Eiro and Heinonen [45], wherein the intermolecular copigmentation effect was investigated between anthocyanins (pelargonidin-3-*O*-glucoside, malvidin-3-*O*-glucoside, or cyanidin-3-*O*-glucoside) and phenolic acids (gallic, ferulic, caffeic, rosmarinic, or chlorogenic acid) at pH 3.37. They proved that the degree of hydroxylation affects the copigmentation reaction. Hence, in this study, C3G molecules with two free hydroxyl groups in the B-ring had a longer estimated half-life, but D3G molecules that possessed three free hydroxyl groups in the B-ring had a shorter half-life (Figure 3). In the case of cyanidin derivatives, it seems likely that the disaccharidic form (rutinoside) of cyanidin exhibited a shorter half-life than its monoglucosidic analog (glucoside), as suggested by Mazza and Brouillard [50]. 

Similar to pH 3.0 samples, D3R appeared to be the most dominant anthocyanin in all samples at pH 6.0 on the day of preparation, with no significant difference between D3G (~23.5%) and C3R (~23.2%), while C3G was the lowest (~19.5%). During storage at 20 °C, C3G showed the highest stability (*p* < 0.05) in all samples with an estimated half-life of 5.5 to 9.4 days. There was no significant difference between D3G, D3R, and C3R in caffeic and control samples, but C3R was slightly higher (*p* < 0.05) than D3G and D3R in both the chlorogenic and rosmarinic acid samples.

Furthermore, Welch et al. [55] reported that anthocyanins can also be degraded by certain oxidative mechanisms, such as the action of the enzyme PPO, which is responsible for the browning of juices from blueberries, strawberries, grapes, and cherries. Jaiswal et al. [56] reported that the oven-drying of fresh pomegranate arils (*Punica granatum* L.) at 90 °C resulted in a 68% loss of PPO activity, suggesting that the drying process did not fully denature or inactivate the PPO enzyme. Moreover, drying processes that kept the moisture content at between 6 to 11% were only capable of minimizing browning reactions of enzymatic and non-enzymatic origin [57]. The moisture content for DBP in this study ranged between 7.62% and 8.77% (*w*/*w*) [58]; hence, there might be some enzyme activity in the pomace. Most of the PPOs studied showed an optimum activity between pH 4 and pH 7 [59]. This is among the reasons why the copigmentation at pH 6.0 in this study showed a lower anthocyanin half-life than at pH 3.0. 

**Figure 5:**
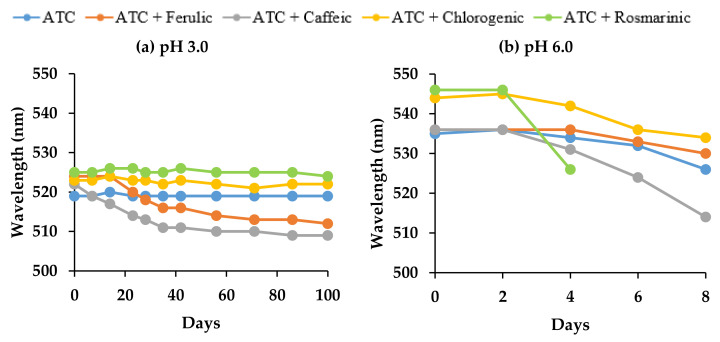
Effect of intermolecular copigmentation on maximum absorption wavelength (λ_max_) (hyperchromic shift) throughout storage in buffer solutions at (**a**) pH 3.0 and (**b**) pH 6.0. *Anthocyanins (ATC).

However, PPO cannot oxidize anthocyanins on its own. Another substrate with *o*-diphenolic compounds (e.g., caffeic, ferulic, and chlorogenic acid) must be present for the first step of polyphenolic oxidation, in which the acid is oxidized into its *o*-quinone form. Then, the anthocyanin with an *o*-diphenolic B ring (cyanidin and delphinidin) is degraded by the *o*-quinone form of acid and forms brown polymers [60]. It is likely that at 20 °C, the PPO action was responsible for the decrease in anthocyanin stability at pH 6.0 (Table 2b), as well as for the change in the copigment complex color to yellowish/orange tonalities in the rosmarinic and caffeic acid samples, compared to the control. This was also indicated by the rapid decrease in the wavelength (Figure 5b). Moreover, it is worth noticing that the increase in copigment concentration at pH 6.0 compared to pH 3.0 not only increased the hyperchromic effect but also may have led to the production of larger aggregates and precipitates, which can affect optical and spectrophotometry behavior [18]. However, in this study, the hypothesis regarding the action of the PPO enzyme is not supported by experimental data.

Other than that, potassium sorbate is commonly used as a preservative in food products due to its antimicrobial properties. In this study, potassium sorbate was added to both the control and copigmentation samples. However, the presence of potassium sorbate most likely resulted in faster degradation of the anthocyanins in the copigmentation samples compared to the control. Moldovan and David [61] investigated the storage stability of anthocyanins from Cornelian cherry fruits (*Cornus mas* L.) at different temperatures (2 °C, 25 °C, and 75 °C) at pH 3.02, in the presence of sodium benzoate and potassium sorbate. Higher stability was observed in the anthocyanin extract without any added preservatives, followed by sodium benzoate, whilst potassium sorbate was the lowest. This indicated that the addition of preservatives interrupted the anthocyanin self-association, leading to their degradation.

#### 3.2.2. Effect on Color Stability

**Figure 6:**
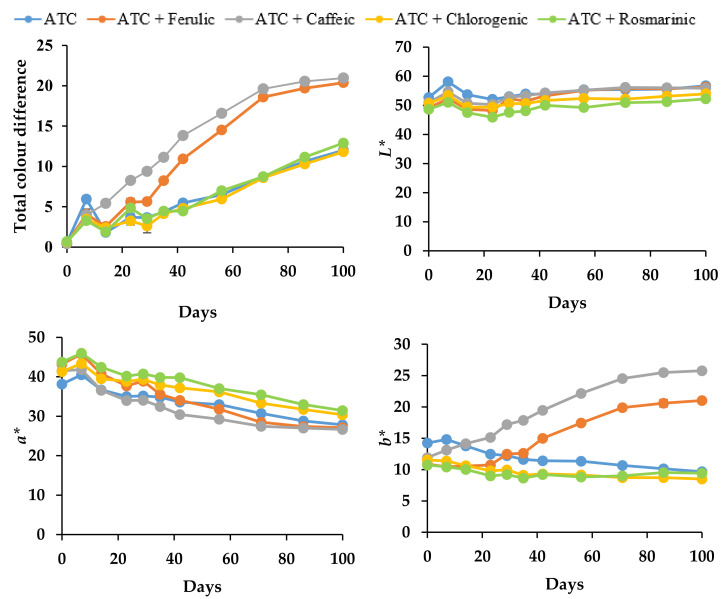

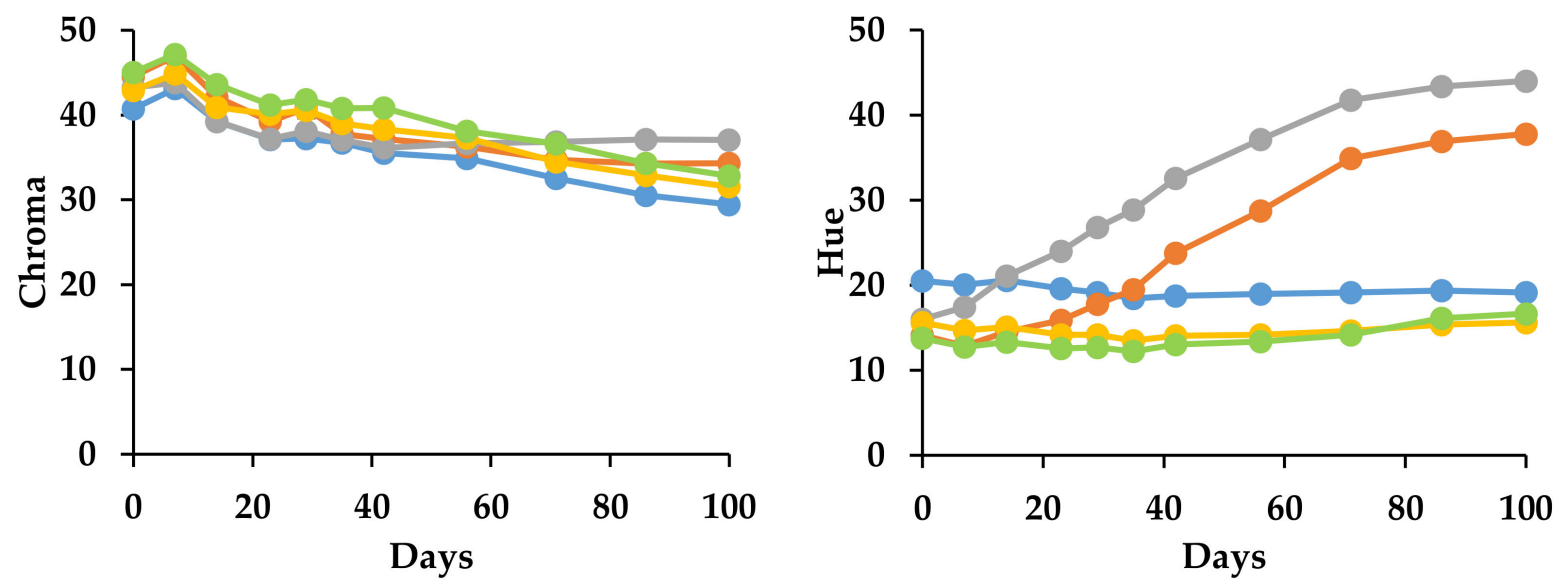
Effect of intermolecular copigmentation on total color difference, L*, a*, b*, chroma, and hue angle during storage at 20 °C in pH 3.0 buffer solutions. *Anthocyanins (ATC); L* (lightness); a* (redness/greenness); b* (yellowness/blueness).

**Figure 7:**
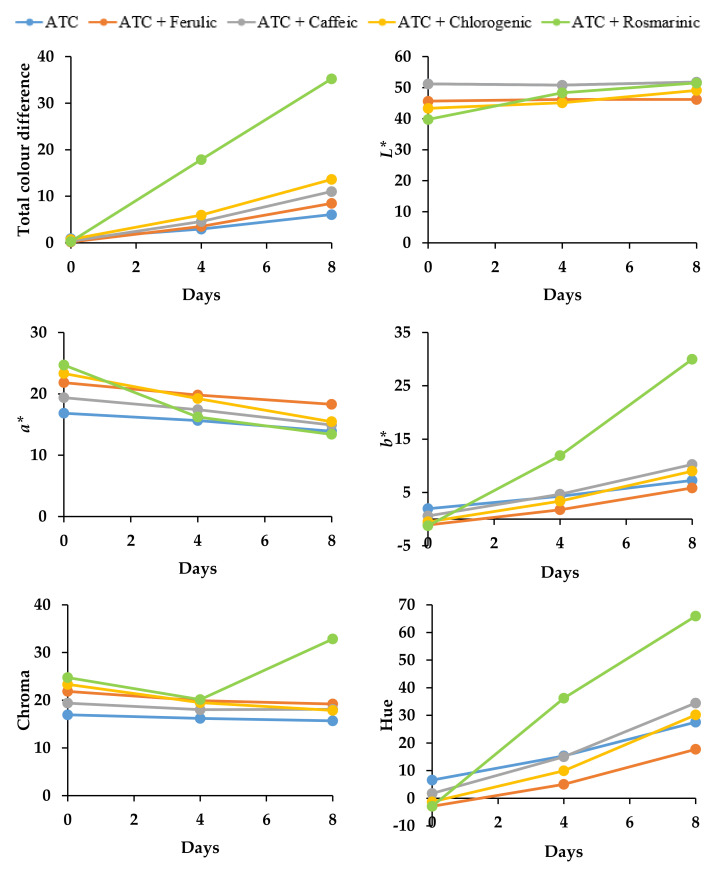
Effect of intermolecular copigmentation on total color difference, L*, a*, b*, chroma, and hue angle during storage at 20 °C in pH 6.0 buffer solutions. *Anthocyanins (ATC); L* (lightness); a* (redness/greenness); b* (yellowness/blueness).

A high percentage of color retention, measured by UV-Vis spectrophotometry, reflects the high color stability of the anthocyanin mixture and consequently low color degradation over time. The changes in the color of the samples were expressed in terms of color values (L*, a*, b*) by Hunter Lab. In addition, a high chroma value indicates a high color intensity, which is accompanied by an increase in absorbance in the visible range (hyperchromic shift) and a shift in the wavelength of the maximum absorbance toward higher values (bathochromic shift) [62]. On day 0 in pH 3.0 and 6.0, copigmentation with rosmarinic acid appeared to have a significant (*p* < 0.05) impact on the color of the solutions. In particular, the L* (darker color) and b* (increase in blueness) values decreased, whereas the a* (decrease in redness) and chroma values increased, indicating the enhancement of color intensity (Figure 6 and Figure 7). 

After 30 days of storage at pH 3.0 (Table 3a), anthocyanins with chlorogenic acid demonstrated better color retention (~86.3%) (*p* < 0.05), indicating higher color stability, with lower total color difference, L*, b*, and hue angle values, as well as higher a* and chroma values (Figure 6), followed by rosmarinic acid (~84.8%) and the control samples (~82.8%). After 90 days, even though chlorogenic acid and the control indicated higher color retention than rosmarinic acid, clearly, chlorogenic and rosmarinic acids had significantly higher chroma (color intensity) (*p* < 0.05) than in the control sample.

Except for taking ferulic and caffeic acids as copigments, a strong correlation (R^2^ = 0.982, *p* < 0.05) was seen between the anthocyanin half-life and color retention over 100 days of storage, suggesting that the anthocyanin content strongly correlated with color stability. In the case of caffeic and ferulic acids, however, even though, after 90 days, a high percentage of color retention was seen in the UV-Vis spectrum (Table 3a), i.e., ~75.2% for ferulic and ~63.2% for caffeic acid, there was a low estimated anthocyanin half-life. The high color retention was probably due to the red-colored form of anthocyanins that transform into yellow chalcone, which led to the yellowish/orange tonalities. This is supported by the hyperchromic shift seen in Figure 5a and the significantly higher (*p* < 0.05) *b and hue angle values in Figure 6, compared to the other copigments. Similarly, Qian et al. [53] determined the effects of gallic, ferulic, and caffeic acids on the anthocyanin stability and color intensification in pH 3.2 buffer, undergoing an accelerated stability test at 95 °C for 15 h. They reported that except for gallic acid, the addition of ferulic or caffeic acid accelerated the degradation of anthocyanins compared to the control but still indicated high color retention. This suggested that the formed copigment complexes did not protect anthocyanins from thermal degradation. Moreover, a computer simulation by Qian et al. [53] proved that the distance from the aromatic surface of gallic acid to the anthocyanin chromophore of peonidin-3-sophoroside-5-glucoside was much shorter (4.37 Å) than the ferulic (4.59 Å) and caffeic acids (5.02 Å), suggesting that gallic acid might protect the anthocyanidin nucleus from hydration better than ferulic and caffeic acids. However, further studies using computational modeling and NMR spectroscopy with pure compounds are needed to fully understand the interactions of the abovementioned co-pigments with anthocyanins.

At pH 6.0, rosmarinic acid was found to greatly influence the color stability, whereas copigmented anthocyanins produced a yellowish/orange color relativity quickly during storage, starting from day 2, as shown in the rapid degradation of the bathochromic shift from 546 to 526 nm (Figure 5b). This is further supported by the significant correlation (*p* < 0.05) between the anthocyanins’ half-life and their color retention (R^2^ = 0.961, *p* < 0.05). A significant (*p* < 0.05) increase in b* and hue angle values (Figure 7) was also observed as a result of the yellow tonalities. Ferulic acid and control samples indicated higher color retention than other copigments; however, ferulic acid obviously had significantly higher (*p* < 0.05) chroma (color intensity) than the control sample.

#### 3.2.3. Effect on Antioxidant Capacity

**Figure 8:**
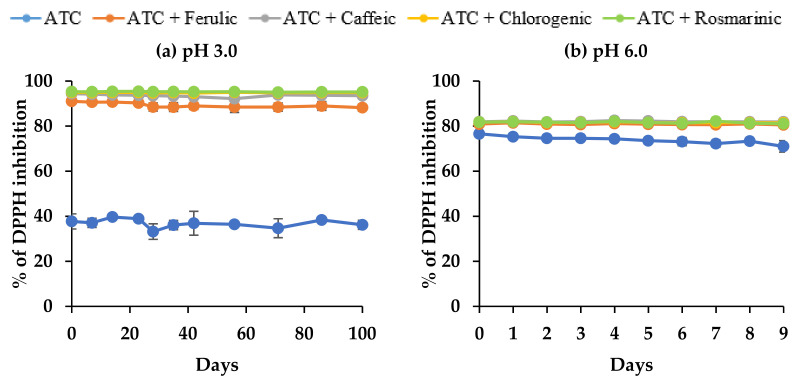
Antioxidant activities of anthocyanins from DBP extracts with phenolic acids as copigments during storage, as measured by a DPPH assay.

Percentage of DPPH scavenging activities in the control sample was significantly higher (*p* < 0.05) (~76.6%) at pH 6.0 compared to pH 3.0 (~39.7%) (Figure 8). This suggested that the pH had a strong influence on the antioxidant capacity of the anthocyanin solutions, where an increase in the pH resulted in an increasing trend of antioxidant capacity. Similar findings were reported by Sui et al. [26] in a comparison between the antioxidant capacity of anthocyanins consisting of C3G and C3R in the different pH of buffer solutions (pH 2.2, 3.0, 4.0, 5.0, and 6.0), where pH 6.0 showed significantly higher antioxidant capacity (0.71 mg Trolox equivalents/mL) than at pH 3.0 (0.45 mg Trolox equivalents/mL).

In the copigmentation reaction, the percentage of DPPH scavenging activities in all samples with copigments increased significantly ((*p* < 0.05); ~95.4% and ~82.3% at pH 3.0 and pH 6.0, respectively). These results are predictable since the added copigment itself is a phenolic acid with antioxidant activity. The significantly higher antioxidant capacity at pH 3.0 compared to pH 6.0 reflected the storage stability of anthocyanins in the copigment samples. Overall, scavenging activity with copigments was stable in all samples during storage at both levels of pH, even though the anthocyanins showed some degradation. This indicated that the degradation products of anthocyanins (phenolic acids and aldehyde) also contributed to the DPPH scavenging activity. Similar findings were reported by Seeram et al. [63] and Terefe et al. [64], where the anthocyanin degradation products (protocatechuic acid, phloroglucinaldehyde, and 4-hydroxybenzoic acid) after the heat treatment of tart cherries assisted in keeping the antioxidant capacity at similar levels. However, further studies are required to confirm any degradation of copigments during storage.

## 4. Conclusions 

Factors such as temperature, pH, copigment type, and enzyme activity substantially influenced the copigmentation complex of DBP anthocyanin solutions and also resulted in color changes. In this study, rosmarinic acid was superior to other phenolic acids in terms of color enhancement at pH 3.0 and pH 6.0 at day 0 but failed to maintain anthocyanin stability during storage at 20 °C. Chlorogenic and ferulic acids were considered to be the best copigments, with the longest estimated anthocyanin half-life at pH 3.0 and pH 6.0 of buffer solutions, respectively. Meanwhile, caffeic acid proved to be the weakest copigment for both pH values. C3G showed the highest stability compared to other individual anthocyanins, due to the suitability of the anthocyanin structure for copigmentation. Other than with the chlorogenic and ferulic acids, anthocyanin self-association was stronger than the intermolecular copigmentation with other phenolic acids. As a result, anthocyanin degradation was faster than color-fading, due to self-association. 

Overall, maintaining anthocyanin copigmentation reactions at low temperatures within cool and dark environments is recommended, especially in a high pH environment, in order to assure the stability of color in the products. Anthocyanins from DBP, copigmented using food-grade chlorogenic acid or ferulic acid, could be considered applicable as natural colorants in low pH foods (jelly) and in high pH foods (ice cream and dairy products). An increase in the ratio of copigments to anthocyanins is also recommended to produce a stronger effect of copigmentation. Overall, it is obvious that copigmentation has an advantage in increasing the anthocyanins’ half-life and the color intensity, as well as stabilizing the antioxidant capacity of products during extended storage.

## Figures and Tables

**Table 1:** Effect of intermolecular copigmentation on the maximum absorption wavelength (λ_max_) and maximum absorbance (*A*_max_) of anthocyanin solutions of DBP extracts with phenolic acids as copigments at Day 0 (copigment:anthocyanin molar ratio of 5:1).

Samples	(a) pH 3.0			(b) pH 6.0		
	λ_max_ (nm)	*A*_max_	∆*A* (%)	λ_max_ (nm)	*A*_max_	∆*A* (%)
ATC	519	0.6189	–	535	0.2641	–
ATC + Ferulic	524	0.7263	17.4 ± 0.4 ^b^	536	0.3484	31.9 ± 1.5 ^c^
ATC + Caffeic	522	0.6664	7.7 ± 0.2 ^c^	536	0.3098	17.3 ± 0.7 ^d^
ATC + Chlorogenic	523	0.6632	7.2 ± 0.1 ^c^	544	0.3956	49.8 ± 1.9 ^b^
ATC + Rosmarinic	525	0.7438	20.2 ± 0.3 ^a^	546	0.4591	73.8 ± 2.1 ^a^

Absorbance (*A*); absorbance increase (∆*A*); anthocyanins (ATC). Values with the same letter ^a,b,c,d^ in each column are not significantly different (*p* > 0.05).

**Table 2:** Effect of intermolecular copigmentation on the estimated half-life time values (t_½_, days) of anthocyanins from DBP extracts, with phenolic acids acting as copigments during storage at 20 ℃ in (a) pH 3.0 and (b) pH 6.0 buffer solutions, as measured by HPLC.

(a) pH 3.0	ATC			ATC + Ferulic			ATC + Caffeic			ATC + Chlorogenic			ATC + Rosmarinic		
	k	t_1/2_ (days)	R^2^	k	t_1/2_ (days)	R^2^	k	t_1/2_ (days)	R^2^	k	t_1/2_ (days)	R^2^	k	t_1/2_ (days)	R^2^
D3G	0.230	51.2 ± 0.7 ^Ba^	0.991	0.558	21.9 ± 0.5 ^Bc^	0.999	0.618	18.2 ± 0.2 ^Bd^	0.989	0.231	50.4 ± 0.8 ^Ba^	0.990	0.258	46.3 ± 1.0 ^Bb^	0.994
D3R	0.303	61.8 ± 1.3 ^Ba^	0.993	0.868	22.7 ± 0.1 ^Bd^	0.999	0.986	18.4 ± 0.1 ^Be^	0.988	0.319	57.9 ± 0.6 ^Bb^	0.995	0.377	52.5 ± 0.3 ^Bc^	0.996
C3G	0.082	208.2 ± 21.4 ^Aa^	0.991	0.192	90.5 ± 4.2 ^Ab^	0.997	0.184	90.6 ± 1.6 ^Ab^	0.994	0.085	216.1 ± 44.0 ^Aa^	0.990	0.077	221.8 ± 3.5 ^Aa^	0.987
C3R	0.168	49.0 ± 1.3 ^Ba^	0.992	0.455	20.0 ± 0.1 ^Bc^	0.998	0.476	16.3 ± 0.1 ^Bd^	0.999	0.167	49.1 ± 0.0 ^Ba^	0.995	0.198	46.0 ± 0.5 ^Bb^	0.999
Total ATC	0.777	71.8 ± 1.8 ^a^	0.994	2.065	28.3 ± 0.1 ^c^	0.999	2.346	22.9 ± 0.2 ^d^	0.994	0.781	71.0 ± 0.1 ^a^	0.995	0.877	66.1 ± 0.9 ^b^	0.995

**(b) pH 6.0**	**ATC**			**ATC + Ferulic**			**ATC + Caffeic**			**ATC + Chlorogenic**			**ATC + Rosmarinic**		
	**k**	**t_1/2_ (days)**	**R^2^**	**k**	**t_1/2_ (days)**	**R^2^**	**k**	**t_1/2_ (days)**	**R^2^**	**k**	**t_1/2_ (days)**	**R^2^**	**k**	**t_1/2_ (days)**	**R^2^**
D3G	2.252	4.7 ± 0.1 ^Ba^	0.996	2.315	4.4 ± 0.1 ^Ca^	0.995	2.817	3.2 ± 0.1 ^Bc^	0.998	2.365	3.8 ± 0.1 ^Cb^	0.991	3.424	1.6 ± 0.1 ^Cd^	0.981
D3R	3.229	5.0 ± 0.1 ^Ba^	0.995	3.044	5.2 ± 0.3 ^Ba^	0.997	4.489	3.1 ± 0.1 ^Bc^	0.996	3.550	4.0 ± 0.1 ^Cb^	0.993	5.516	1.7 ± 0.1 ^Cd^	0.984
C3G	0.920	9.1 ± 0.6 ^Aa^	0.996	0.853	9.6 ± 0.1 ^Aa^	0.998	1.158	6.8 ± 0.2 ^Ab^	0.995	0.886	8.9 ± 0.2 ^Aa^	0.994	1.257	5.5 ± 0.1 ^Ac^	0.992
C3R	2.067	4.9 ± 0.1 ^Bb^	0.996	1.709	5.7 ± 0.1 ^Ba^	0.994	3.218	2.9 ± 0.1 ^Bd^	0.985	2.032	4.7 ± 0.1 ^Bc^	0.994	3.253	2.4 ± 0.1 ^Be^	1.000
Total ATC	8.459	5.3 ± 0.1 ^a^	0.996	8.053	5.5 ± 0.1 ^a^	0.997	11.158	3.6 ± 0.1 ^c^	0.996	8.833	4.6 ± 0.1 ^b^	0.994	12.577	2.3 ± 0.1 ^d^	0.989

Estimated half-life time (t_½_); zero order reaction rate constants (k); correlation coefficient (R^2^); anthocyanins (ATC). Values with the same letter ^a,b, c,d^ in each column are not significantly different (*p* > 0.05). Values with the same letter ^A, B, C,D^ in each row are not significantly different (*p* > 0.05).

**Table 3:** Effect of intermolecular copigmentation on color range retention during storage at 20 °C, at pH 3.0.

(a) pH 3.0	ATC	Ferulic	Caffeic	Chlorogenic	Rosmarinic
Day 0	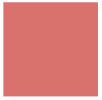	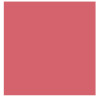	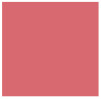	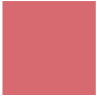	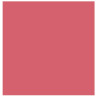
Day 30	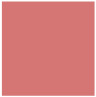	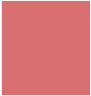	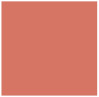	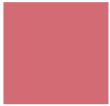	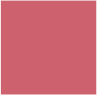
Color Ret (%) at Day 30	82.8 ±0.5 ^bc^	79.8 ± 0.3 ^d^	81.7 ± 0.3 ^cd^	86.3 ± 1.3 ^a^	84.8 ± 0.4 ^ab^
Day 90	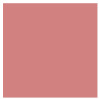	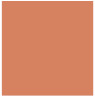	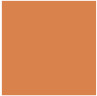	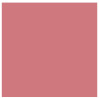	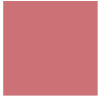
Color Ret (%) at Day 90	60.0 ± 0.3 ^c^	63.2 ± 0.4 ^b^	75.2 ± 0.0 ^a^	60.9 ± 0.4 ^c^	57.8 ± 0.9 ^d^

(b) pH 6.0	**ATC**	**Ferulic**	**Caffeic**	**Chlorogenic**	**Rosmarinic**
Day 0	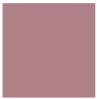	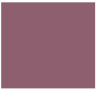	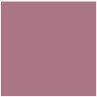	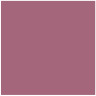	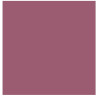
Day 4	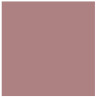	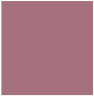	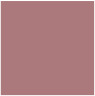	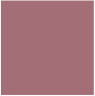	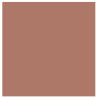
Color Ret (%) at Day 4	94.1 ± 0.3 ^a^	93.1 ± 0.2 ^a^	88.7 ± 0.0 ^b^	86.4 ± 1.3 ^b^	60.2 ± 1.4 ^c^
Day 8	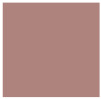	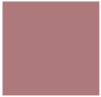	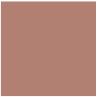	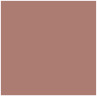	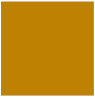
Color Ret (%) at Day 8	84.5 ± 1.8 ^a^	82.6 ± 0.1 ^ab^	76.2 ± 2.0 ^b^	64.5 ± 0.4 ^c^	48.5 ± 2.6 ^d^

Conversion of L*, a*, and b* values to RGB (red, green, blue) color model for visualization of anthocyanin color (http://www.easyrgb.com/en/ accessed on 20 June 2022). Anthocyanins (ATC). Values with the same letter ^a,b,c,d^ in each column are not significantly different (*p* > 0.05).
